# Metabolic regulatory oscillations in intertidal green seaweed *Ulva lactuca* against tidal cycles

**DOI:** 10.1038/s41598-017-15994-2

**Published:** 2017-11-27

**Authors:** Vishal Gupta, Hemant R Kushwaha

**Affiliations:** 1 Biological Oceanography Division, CSIR-National Institute of Oceanography, Goa, 403004 India; 2 School of Biotechnology, Jawaharlal Nehru University, 110067 New Delhi, India

## Abstract

The survival of wetland plant species largely relies on physiological adaptations essential for submergence and desiccation. Intertidal seaweeds, unlike terrestrial plants, have unique adaptations to submergence and can also sustain desiccation arising from tidal rhythms. This study determined the differential metabolic regulations in the inter-tidal seaweed species *Ulva lactuca* against the submergence and desiccation. During desiccation, the relative water content of the algal thalli declined with concomitant increase in reactive oxygen species (ROS) and lipid peroxidation. Nevertheless, the trends reversed during recovery on re-submergence and attained homeostasis. Metabolite profiling of *U*. *lactuca* revealed desiccation induced balance in energy reserve utilization by adjusting carbohydrate metabolism and switch over to ammonia metabolism. Upon re-submergence, thalli showed an increase in fermentative metabolites, pyruvate-alanine conversion, and the GABA shunt. Prolonged submergence induced substrate level phosphorylation mediated sugar biosynthesis while continuing the alternative carbon flux through fermentative metabolism, an increase in osmoprotectants glycine and betaine, sulfur bearing compounds cysteine and hypotaurine, and phenolic compound coniferaldehyde. The determined metabolic regulations in *U*. *lactuca* for submergence tolerance provide insights into potential evolutionarily conserved protective mechanisms across the green lineage and also highlights the possible role of sulfur oxoforms as strong free radical scavengers.

## Introduction

Marine macroalgae (seaweeds) represent the earliest diverging and evolutionary diverse aquatic organism occupying the basal position in the aquatic food web of intertidal and subtidal regions^1^. This group of aquatic plants have the unique physiology of being able to grow in fully submerged conditions representing the contrasting homeostasis regulations over land plants. Seaweeds, with their unique adaptability to submerged conditions make them a potential candidate to investigate the regulation of this trait. Seaweed species of the genus *Ulva* are most dominant in the upper intertidal zone where they regularly experience submergence and desiccation cycles with tides. The natural adaptation of *Ulva* species to submergence and desiccation therefore represents the existence of a unique homeostasis regulation. Furthermore, unlike terrestrial crops, *Ulva* has simple diastromatic thallus without any complex cellular level organization. This implies that the adaptive regulations are mostly cellular responses as opposed to complex anatomic modification in other plants to tolerate submergence. Earlier studies on understanding the homeostasis in *Ulva* were restricted to biochemical responses against the stress of irradiance^2–4^ salinity and temperature^5^ arising from desiccation in the intertidal region. The natural adaptability of this group of organisms to submergence is not been analysed.

In land plants, the prime downstream signalling regulator of submergence stress is ethylene biosynthesis^6^ which is presumed to be lost in marine plants such as seagrasses while their secondary adaptation to aquatic habitat^7^. This, therefore implies a unique metabolic regulations in marine plants or in seaweeds for maintaining their submerged physiology. The unavailability of extensive large-scale genomics data is the major bottleneck towards understanding the regulations in *Ulva* against acute stresses of intertidal zonation. Nevertheless, the differential expression of metabolites through untargeted metabolomics approach may provide insights into the possible underlying regulatory mechanisms.

The untargeted metabolomics may facilitate the functional annotations of a particular physiological state of an organism in the absence of whole genome information^8^. The metabolome analysis facilitates the determination of cell’s catalytic and regulatory processes. Further, metabolome represents the immediate biochemical consequences of genomic and transcriptomic activity and hence holds more biological relevance than other ‘-omes’^9^. In terrestrial life forms, metabolomics has made significant contributions towards investigating the responses to environmental stress, cues for biomarkers, chemotaxonomy, mutant differentiation and comparing growth stages, drug discovery, studying global effects of genetic manipulation, and natural product discovery^10–13^. Despite various advantages of metabolomics approach, studies are far behind in seaweeds compared to land plants. To date, no report describes the metabolic regulatory transitions happening during desiccation and submergence cycle in seaweeds. The present study is an attempt to understand the unique adaptation of *Ulva lactuca* tolerating periodic desiccation-submergence cycle using a holistic approach of untargeted metabolomics. Thus, a ‘learn from nature approach’ using a model organism naturally adapted for submergence and exposure, such as seaweed, can be supportive in understanding the submergence tolerance mechanisms either evolutionarily conserved or unique.

## Results

### Relative water content (RWC %)

**Figure 1:**
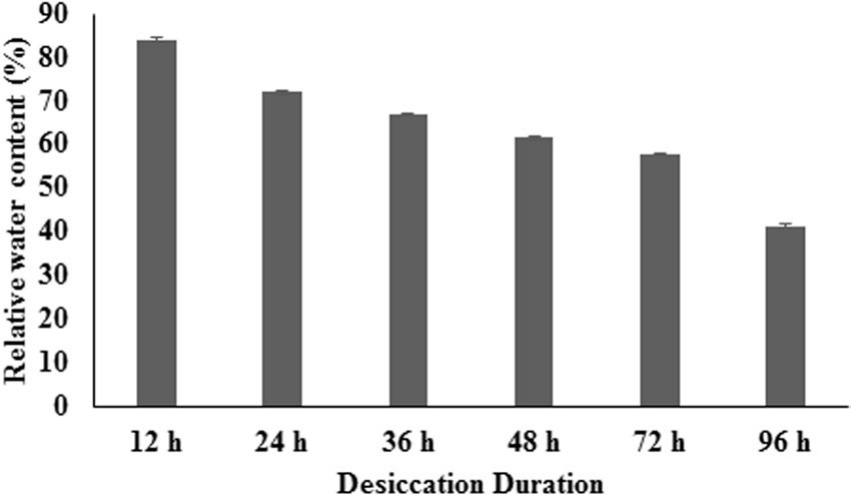
Desiccation induced relative water content (%) for seaweed species *U*. *lactuca*.

Desiccation stress caused a significant decrease in the RWC. The RWC showed a marginal decline of 16% on desiccation exposure for 12 h which declined significantly with further increase in desiccation durations. The RWC declined to 28%, 33%, 38%, 42% and 58% in the thalli desiccated for 24, 36, 48, 72 and 96 h respectively (Fig. 1). On re-submergence after the desiccation time points of 12, 24, 36, 48 and 72 h, algae could able to recover and grow normally. This indicates that the RWC decline to less than 50% allow *U*. *lactuca* to recover on submergence. However, the thalli desiccated for 96 h lost more than 50% of RWC were not able to survive when rehydrated back in natural seawater.

### Lipid peroxidation and ROS determination

**Figure 2:**
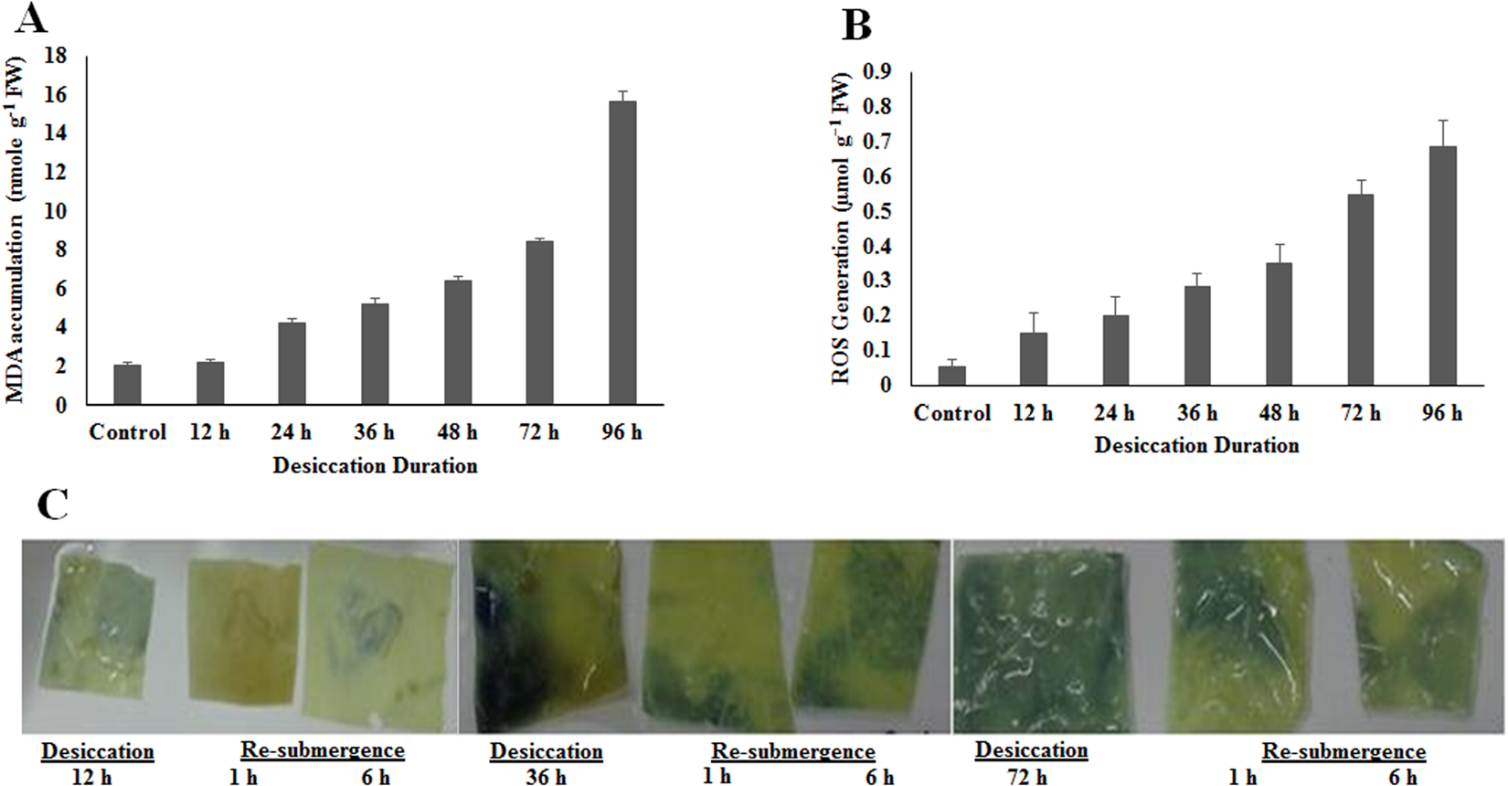
Desiccation induced change in (**A**) lipid accumulation, (**B**) generated reactive oxygen species (ROS). (**C**) Schematic images of histochemical localization of O_2_^•−^ by NBT staining after desiccation and re-submergence.

The decrease in the RWC on desiccation was accompanied by an increase in lipid peroxidation and ROS generation in the thalli (Fig. 2A). The increase in lipid peroxidation was determined from an increased absorbance for malondialdehyde (MDA in nmol g^−1^ FW). The MDA accumulation showed a linear increase with the increase in desiccation duration. MDA level showed a slight increase of 2.21 ± 0.18 nmol g^−1^ FW after desiccation of 12 h. Prolonged desiccation for another 12 h showed two fold increase in lipid peroxidation (4.21 ± 0.21 nmol g^−1^ FW). Further extension in desiccation duration led to a significant increase in lipid peroxidation. The lipid peroxidation or MDA accumulation increased to 5.25 ± 0.24, 6.43 ± 0.24, 8.47 ± 0.13, 15.67 ± 0.48 nmol g^−1^ FW after desiccation of 36, 48, 72 and 96 h (Fig. 2A). Subsequent re-submergence reverted the lipid peroxidation levels to normal. The MDA accumulation on re-submergence was in the range of 2.08 ± 0.14 nmol g^−1^ FW to 3.41 ± 0.12 nmol g^−1^ FW for all the time points studied.

ROS generation was found to increase with an increase in desiccation duration (Fig. 2B). The ROS level (µmol g^−1^ FW) was found 0.05 ± 0.02 in control thalli while the ROS level showed ten-fold increase (0.15 ± 0.05 µmol g^−1^ FW) on desiccation for 12 h. The increase in desiccation duration to 24 and 36 h showed a steady increase in ROS levels to 0.20 ± 0.05, 0.28 ± 0.03 µmol g^−1^ FW. Further increase in desiccation period to 48, 72 and 96 h showed a linear-fold change in ROS generation with recorded values as 0.35 ± 0.05, 0.55 ± 0.04 and 0.68 ± 0.08 µmol g^−1^ FW respectively (Fig. 2B). On re-submergence for 2, 6 and 12 h after desiccation period of 12 h, the ROS level was estimated as 0.11 ± 0.05, 0.08 ± 0.04 and 0.07 ± 0.05 µmol g^−1^ FW respectively. The level of ROS remained in the range of 0.04 ± 0.02 to 0.08 ± 0.05 on continued submergence. The decline in ROS level from other desiccation time points were also in similar ranges as observed for desiccation of 12 h. This indicated the recovery potential of algal thalli on re-submergence after desiccation. However, algal thalli desiccated for 120 h on re-submergence did not show any change in ROS accumulation for 2 and 6 h and suddenly showed increase in ROS accumulation which led to decay in algal thalli. The histochemical staining employed to detect *in situ* accumulation of O_2_^•−^ and H_2_O_2_ radicals (two important representatives for ROS) using NBT and DAB respectively further confirmed the accumulation of ROS with the increase in desiccation followed by a decline in their accumulation on re-submergence. A blue formazone formed by reduction of NBT by O_2_^•−^ is clear evidence for the generation of superoxide radical (Fig. 2C). Similarly, the formation of H_2_O_2_ dependent brown precipitates was contingent with the exposure duration and accumulated maximum in the thalli exposed for longer duration.

From these analyses, samples desiccated for 24, 36 and 72 h and their subsequent submergence time points were selected for further analysis of metabolomics.

### Metabolic profile

The untargeted metabolite profile for the seaweed species *U*. *lactuca* was generated using NMR spectroscopy. Metabolites identities were confirmed by both 1 H and 2D NMR approaches. The metabolite profile revealed the abundance of sugars, amino acids, organic acids, osmolytes, phenolic and sulfinic compounds (Table 1). The sugar metabolites identified were mainly the glucose and sucrose. The relative concentration of sucrose was highest among all the metabolites identified. Among amino acids, cysteine and glycine were high while other amino compounds such as choline, GABA, triethanolamine and glutamic acid were low. The fermentative pathway metabolites lactate, formic acid and acetate were also determined in this study. The compounds such as hypotaurine, ascorbate, allantoin, and coniferaldehyde were detected which have prominent anti-oxidative roles.

**Table 1:** Identified metabolites and their signature peak (ppm) values from 1D and 2D NMR spectroscopy.

Metabolites	Chemical Sift δ, ppm	
	^1^H	HSQC (^1^H-^13^C)
Glucose	4.2 (d)	
Sucrose	5.4 (s)	
Lactate	1.33 (d), 4.1 (m)	1.3–22.8; 4.12–71.2
Acetate	1.92 (s)	2.0–26.0
Alanine	1.48 (d), 3.65 (q)	1.35–18.8; 3.8–53.5
Glutamic acid	2.15 (m), 2.4 (m)	2.1–29.48; 2.4–36.22; 3.8–57.5
Hypotaurine	2.72 (t), 3.45 (t)	2.7–58.1; 3.4–36.4
Coniferaldehyde	3.64 (d), 6.85 (bd), 8.5 (s)	3.6–56.4; 7.21–124.2
Glutamine	2.1 (q), 2.43 (m)	3.8–56.8; 2.49–33.92; 2.17–29.0
Cysteine	2.98 (m), 4.01 (dd)	2.98–28.4; 4.01–59.0
Choline	3.19 (s), 3.5 (m)	4.0–59.0; 3.21–54.89; 3.6–58.5
Betaine	3.19 (s), 3.9 (s)	3.19–56.0; 3.89–67.1
Glycine	3.55 (s)	3.5–44.1
Triethanolamine	3.88 (s), 3.4 (s)	3.84–57.8; 3.34–57.8
Formic acid	8.44 (s)	
Allantoin	5.4 (s)	
Succinate	2.3 (s)	
α-ketoglutarate	2.45 (s), 2.5 (s)	

### Metabolic variations during desiccation-submergence cycle

**Figure 3:**
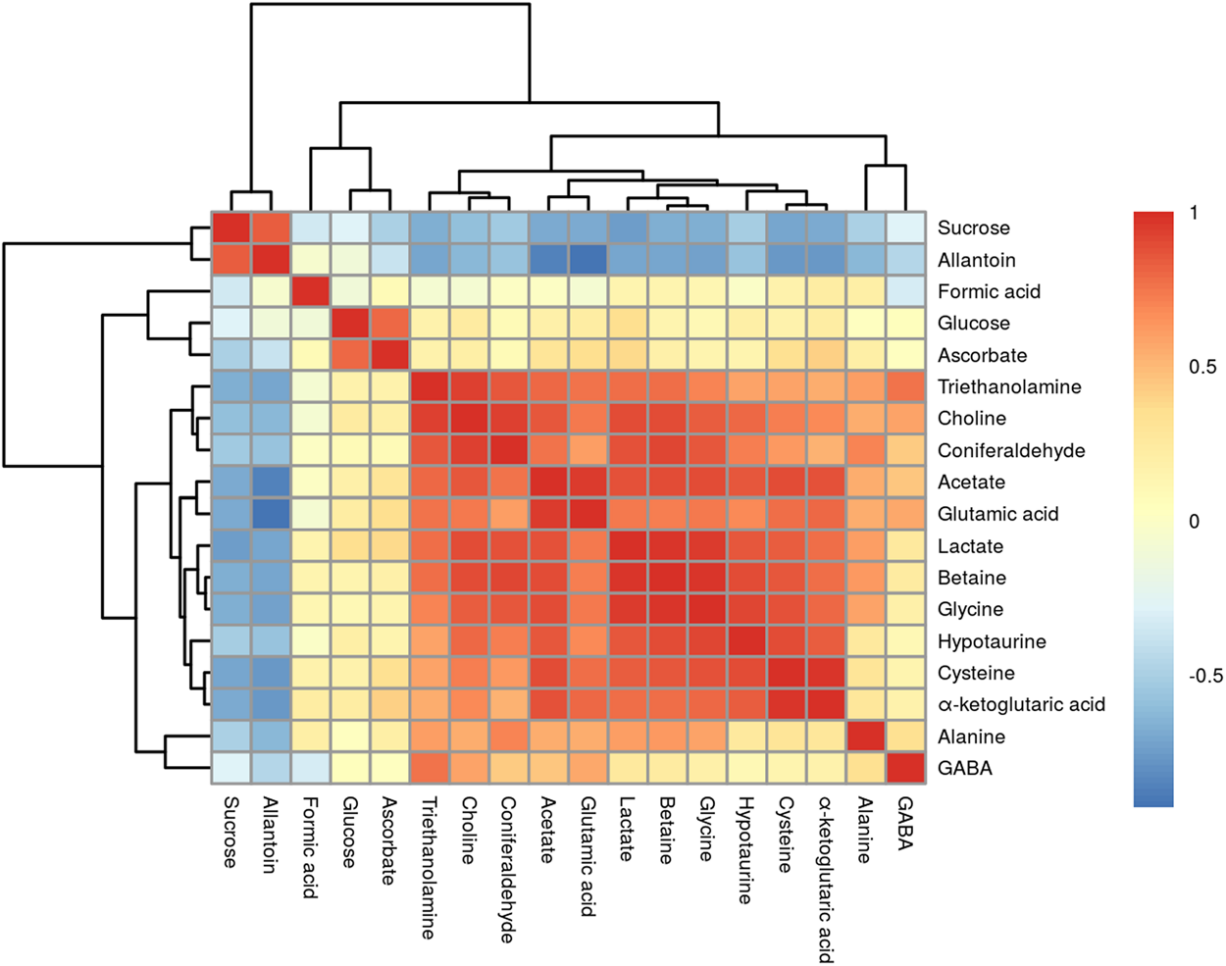
Heatmap representation of the correlation among the identified metabolites during different desiccation and re-submergence time periods.

**Figure 4:**
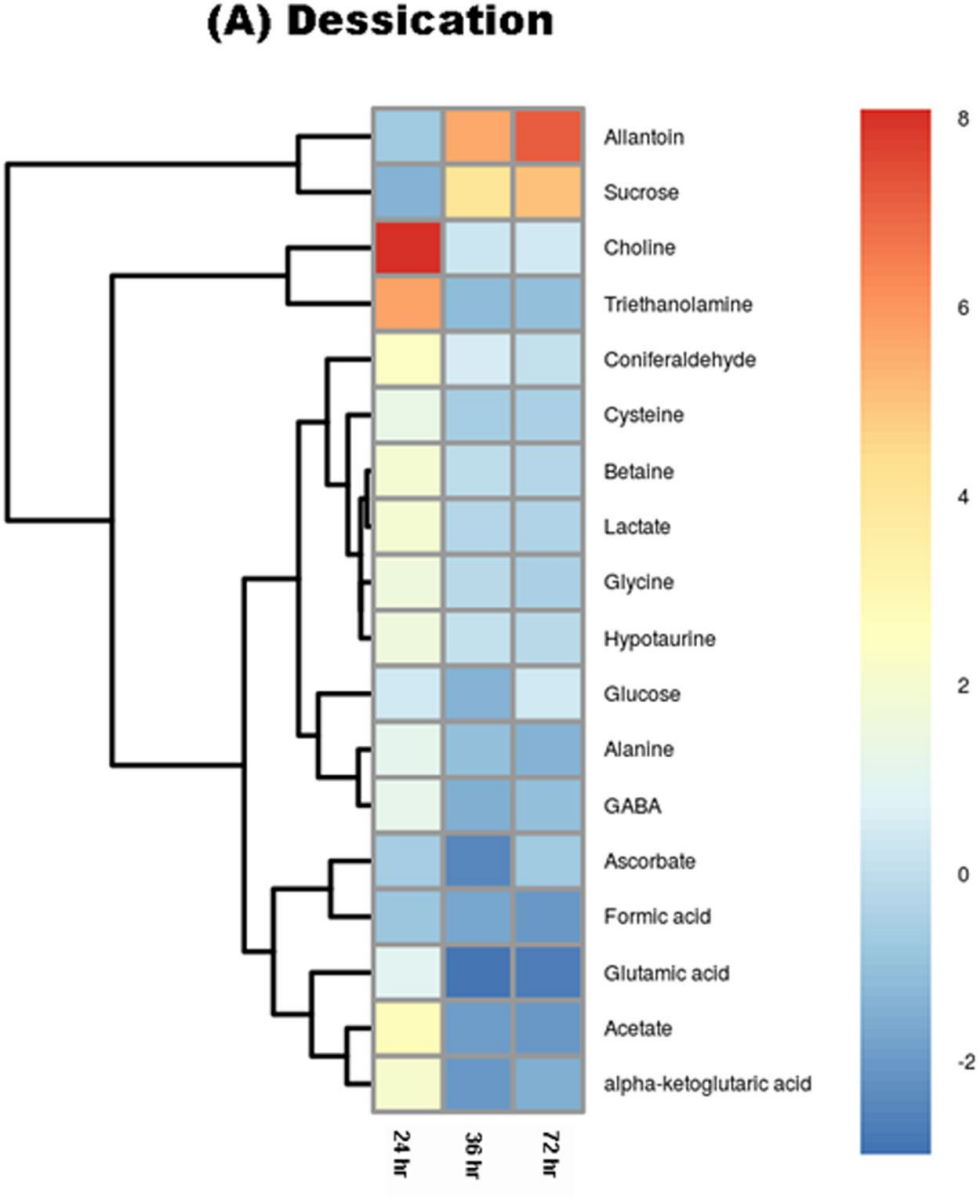
Heatmap representation for fold-change variation of concentration of metabolites at different desiccation periods of 24 h, 36 h and 72 h in comparison to control submerged sample.

The metabolic variations were determined by comparing the normalized values of each metabolite detected at different treatment times. The samples of desiccation point of 24 h, 36 h and 72 h were compared with respect to always submerged control samples to determine the differential expression of metabolite during desiccation stress. All the metabolites identified showed significant correlation in their expression during different experimental conditions of desiccation and submergence (Fig. 3, Supplementary Figure 1). The results revealed a significant increase in all the metabolites except for sugars glucose, and sucrose after 24 h of desiccation. Further increase in desiccation to 36 h and 72 h showed contrasting behaviour with decline in the concentration of most of the identified metabolites except sugars glucose and sucrose (Fig. 4). The decline in the metabolite concentration was congruent to the increase in desiccation duration.

**Figure 5:**
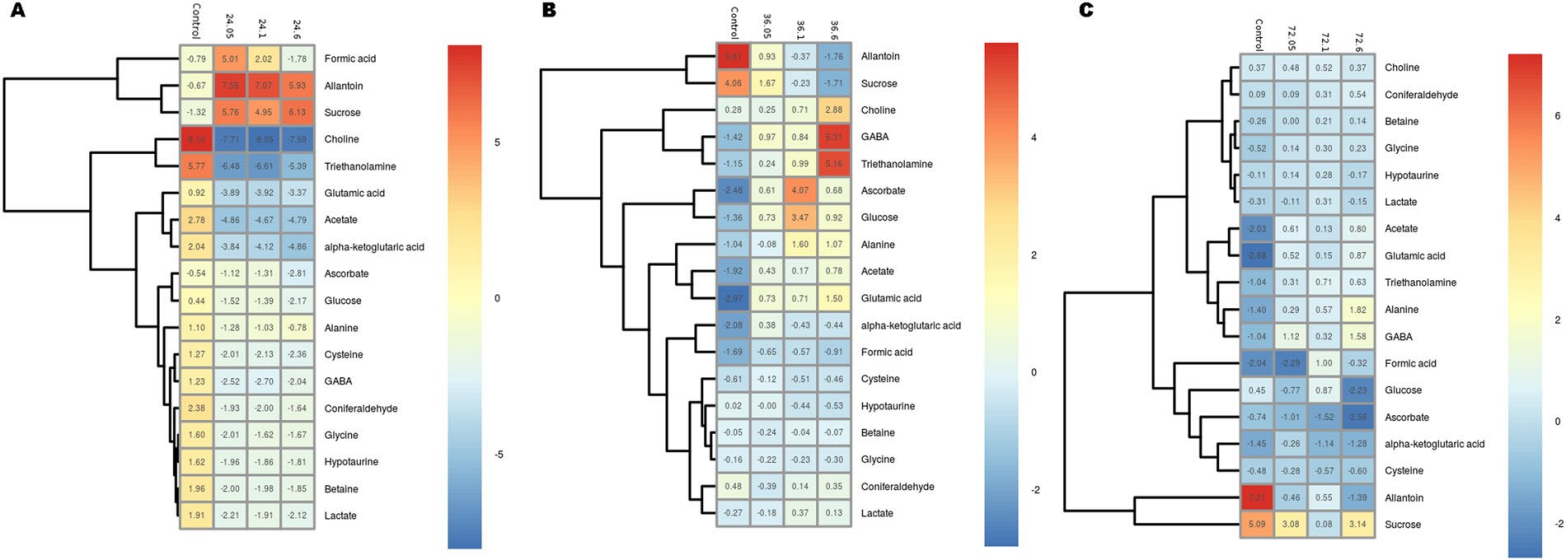
Heatmap representation for fold-change variation of concentration of metabolites at re-submergence of 30 min, 1 h and 6 h after desiccation periods of (**A**) 24 h, (**B**) 36 h and (**C**) 72 h.

After determination of differential metabolite expression during desiccation, metabolite variations were determined in re-submerged samples (0.5 h, 1 h and 6 h) of each desiccation time point (Fig. 5). For this, the comparisons were initially made individually between the desiccation time point and its corresponding re-submergence time periods. The re-submergence of samples after a desiccation period of 24 h revealed a marked decline in the normalized values for all the metabolites detected at all the time points except for sucrose and allantoin (Fig. 5A). The algal thalli re-submerged after desiccation period of 36 h and 72 h showed contrasting metabolites expression compared to the same revealed after 24 h desiccation. The content of acetate, alanine, α-ketoglutaric acid, choline, GABA, glutamic acid, lactate and triethanolamine increased in both the formar cases while allantoin, and sucrose were decreased. Glucose and ascorbate showed contrasting behaviour in both the conditions with an increase on re-submergence after 36 h (Fig. 5B) of desiccation and decrease in the case of re-submergence after 72 h of desiccation (Fig. 5C). Other metabolites did not show significant differences. Further, a comparison of metabolite variation between each re-submergence time point was made. The results revealed increase in the normalized values for alanine, betaine, coniferaldehyde, and lactate, and decline in acetate, choline, GABA, glucose, glutamic acid, hypotaurine and sucrose, while no change in cysteine, glycine and triethanolamine at 0.5 h of re-submergence after desiccation period of 36 h and 72 h over the same after desiccation period of 24 h. Similar trends of metabolic variations were observed for 1 h and 6 h of re-submergence. However, small variations were observed on re-submergence for 6 h after desiccation period of 72 h whereas acetate and alanine declined while glucose, lactate and triethanolamine increased.

## Discussion

The present study provides insights into the underlying metabolic regulations in intertidal seaweed species *U*. *lactuca* against the experienced desiccation and submergence. The cellular level damage by the excess production of ROS is the main source of injury. ROS generation is associated with most stresses of biotic and/or abiotic origin. Earlier studies on biochemical responses of desiccation stress tolerance in seaweeds revealed a marked increase in ROS generation^14–16^. The *U*. *lactuca* in this study could tolerate the RWC loss up to 50% with concomitant increase in ROS generation. The ROS generation and lipid peroxidation increased to the level that caused unrecoverable cellular damage when RWC loss exceeded above the 50%. The other intertidal seaweed species of genus *Pyropia* and/or *Porphyra* is reported to tolerate the water loss up to 90% despite overproduction of ROS and hypothesized to possess unique antioxidant machinery^17^. However, the non-tolerant seaweed species are reported to have major damage with slight misbalance in cellular water content^18,19^. Subsequent to desiccation, the re-submergence of algal thalli showed decline in ROS generation and lipid peroxidation confirming the recovery from the stress. Prolonged, submergence keep the ROS level stagnant which is in the limit of detoxification by normal antioxidative machinery.

Most of the compounds identified are the primary metabolites with known ubiquitous expression in land plants in different physiological conditions. Desiccation showed a significant decline in the concentrations of primary sugars glucose and sucrose reflecting the stress regulation mainly through increased carbohydrate metabolism^18–20^. The recorded decrease in primary sugars in the present study is associated with increase in α-ketoglutaric acid from TCA cycle, pyruvate mediated alanine biosynthesis and amine-group bearing compounds such as glycine, triethanolamine, and glutamic acid. Prolonged desiccation induced metabolic alterations to cease energy reserve by accumulation of sugars, TCA cycle compounds, and N metabolism while downregulating the energy expensive fermentative metabolic pathway compounds. The content of GABA also increased with increase in desiccation. Glycine, triethanolamine, and glutamic acid are regulators for glutathione biosynthesis and hence confirms their antioxidative role to scavenge the increased ROS with the increase in desiccation. Thus, the regulation against desiccation stress mainly includes the energy reserve depletion by increase in carbohydrate metabolism which in turn being switched over to ammonia metabolism through alanine, glutamine, and glutathione based regulatory pathways or photorespiration. Similar trends of metabolic variations were reported in *E*. *siliculosus* against the stress of hypo and hyper-salinity^21^. Accumulation of GABA is reported to be as a putative redox regulatory mechanism in various land plants like *Lotus japonicus* ^5^, *Pisum sativum* ^22^, *Arabidopsis* ^23^ and *Glycine max* ^24^.

The metabolic regulations for submergence tolerance showed important role of metabolites related to the pathways of glycolysis, fermentative and nitrogen metabolism. The increase in fermentative pathway metabolites i.e. acetate, lactate cause increased cellular acidification which is balanced by the increase in alanine, and activation of GABA shunt and glutamate-GABA cycle^20^. The presence of both acetate and lactate is indicative of the existence of PDH bypass which regenerates NAD^+^ and allows the tricarboxylic acid (TCA) cycle to continue^25^. Similar metabolic alterations have been reported from aquatic to land plants experiencing hypoxia on submergence^22–26^. On deprivation of carbon assimilation during submergence, the plants switch their metabolic activity to nitrogen assimilation^27,28^. This mechanism seems to be conserved and reported in unicellular primitive green alga *Selenastrum minutum* ^29^ to land crops such as *Medicago* ^30^ and rice^31^. The increase in glycine induce glutamate:glyoxylate aminotransferase activity was reported as regulators against hypoxia^32–34^. The same phenomenon is evidenced in the present study along with the activation of GABA shunt mediated through TCA cycle intermediate α-ketoglutaric acid. This supports an increases in ATP generation by proton gradient flux yielding energy under hypoxia. The results of the present study are in congruence to the reported hypoxia-induced metabolic regulations in seagrass species *Zostera marina* ^26^ and other plants^32–34^.

The metabolic alterations on submergence after 36 h and 72 h of desiccation were different from the same recorded for submergence after 24 h desiccation. The concentration of acetate, lactate, alanine, GABA and glutamic acid were higher in former two samples. Also, this increase in metabolites continues with the increase in time of submergence. The finding is in congruence to the metabolic regulations reported in higher plants that submergence increased metabolic flux towards fermentative and nitrogen assimilation pathways in response to applied environmental stresses. Interestingly, the rate of accumulation of primary metabolites of substrate level phoshorylation i.e. glucose along with osmoprotectants glycine and betaine were found to increase upon re-submergence after increase desiccation period. Our transcriptomics study revealed upregulation of S-adenosylmethionine synthetase (unpublished), a cofactor in the synthesis of compatible solutes such as glycine and betaine. This is also a major stress regulator from unicellular algae to complex land plant forms^35,36^. The increase in substrate level phosphorylation probably continues the ATP generation thus decrease the dependency over oxidative phosphorylation.

**Figure 6:**
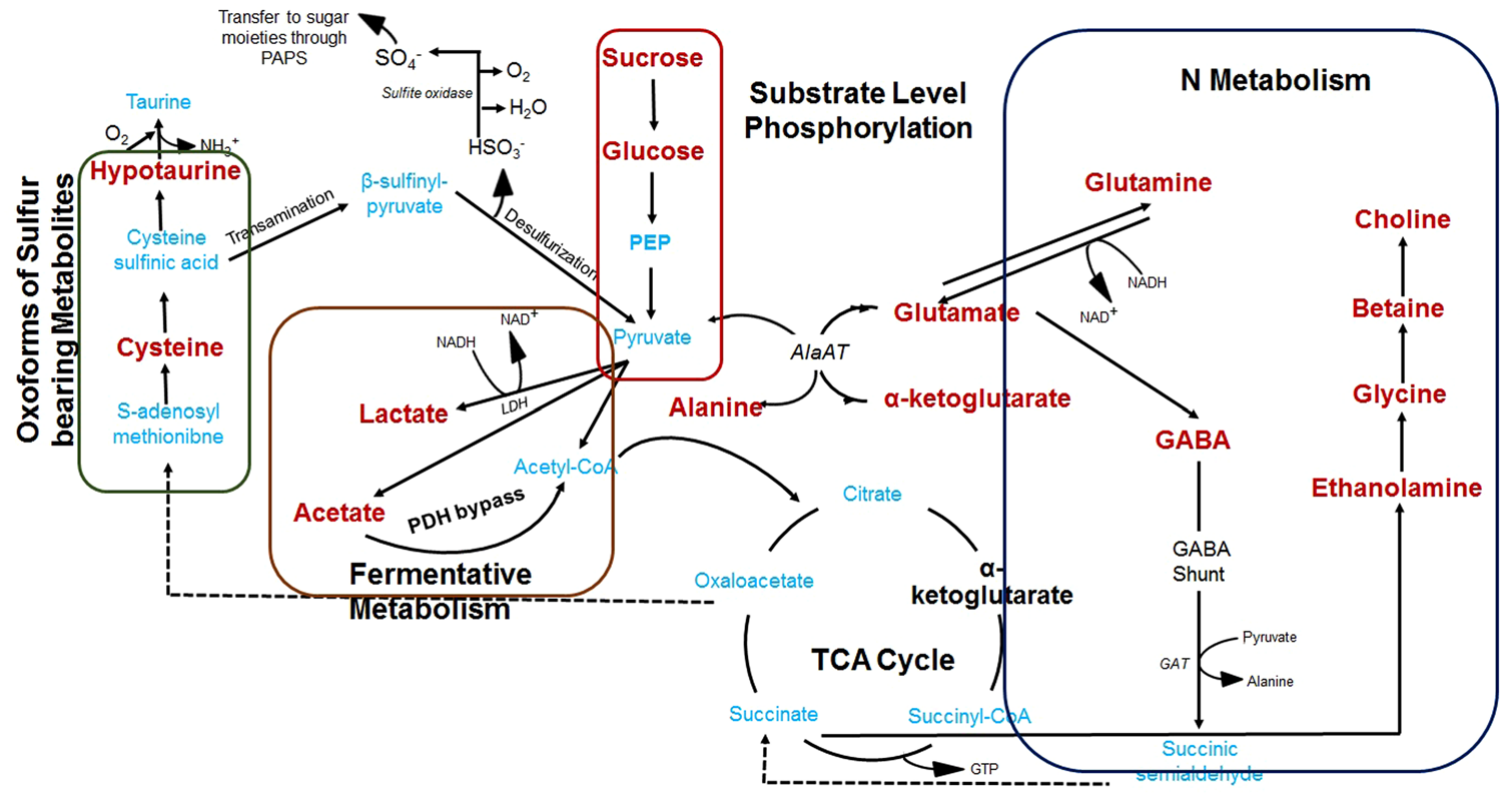
Conceptualized metabolic pathways regulating desiccation and submergence cycle in green seaweed species *Ulva lactuca*.

Interestingly, this study identified sulfur bearing metabolites cysteine and hypotaurine, phenolic compound coniferaldehyde with increased accumulation upon submergence. The former two are considered as non-proteinogenic cysteine-oxoforms having a promising role in free radicle detoxification^37^. The chemical structure of these metabolites make them labile for rapid transitions to different oxidative states eventually making them effective quenchers for free radicles^8,9^. Such metabolites along with cysteinsulfinic acid and isethionic acid were reported by Gupta *et al*.^8,9^ while performing untargeted metabolomics study in different seaweed species. However, their possible role was not determined. This study for the first time provide the evidence for their metabolic role in stress tolerance. A conceptualized model based on the findings from the present study is drawn to provide insights about the regulations seaweeds have to sustain and grow in submerge condition (Fig. 6).

## Conclusion

This study for the first time revealed the differential metabolic regulations in intertidal seaweed species *U*. *lactuca* against desiccation-submergence cycle arising from periodic tidal rhythms. The desiccation stress is regulated by the balance in carbohydrate metabolism and switching to ammonia metabolism. The increase in glycolysis, fermentative and nitrogen metabolism is determined as major regulators to attain homeostasis upon submergence after desiccation. Also, the pyruvate-alanine cycle, and activation of GABA shunt increased with submergence. These regulations are conserved throughout the green lineage from unicellular to complex terrestrial plants. The prolonged submergence showed a distinct homeostatic response in *U*. *lactuca* with accumulation of more sugars along with osmoprotectants glycine and betaine. The fermentative metabolism continues indicating the NAD^+^ generation and the toxic effect of fermentative products is balanced by alternative carbon flux. Thus, seaweed have unique adaptation of energy generation during continuous submergence through substrate level phosphorylation, oxidative phosphorylation and controlled fermentative metabolism. The detailed transcriptome level study may further provide insights about the underlying regulations seaweed have for submergence tolerance unlike terrestrial plants.

## Materials and Methods

### Sample collection

Seaweed samples of species *Ulva lactuca* were collected from Veraval (N 20° 54.87′; E 70° 20.83′) along Gujarat coast, India. The collected thalli were maintained in unialgal culture in the laboratory in sterile enriched seawater media^38^ under white fluorescent lamps of irradiance intensity about 50 µmole photon m^−2^ s^−1^ with a 12: 12 h light:dark photoperiod. The algal thalli were made axenic following the method described by Reddy *et al*.^39^ and subjected to the stress of desiccation followed by submergence.

### Desiccation and submergence treatment

For desiccation treatment, the thalli were exposed to air during light period while keeping their bottom surface in the wet sand (moisture content 10%). For this, the algal thalli were spread on a propylene plastic tray with autoclaved sea sand and incubated at 25 ± 2 °C with relative humidity 65 ± 2%. The desiccation treatment given was for different durations of 12, 24, 36, 48, 72, 96 h. Followed by each desiccation time, algal thalli were re-submerged in autoclaved seawater. After submergence, samples were collected at the periodic interval of 0.5, 1 and  6 h and analysed for their recovery by biochemical and metabolite characterizations.

### Relative water content estimation

The relative water content (RWC) after the desiccation duration was considered as the measure to estimate the recovery of algal thalli on re-submergence. The RWC was calculated as follows: RWC = (LWt −DW)/(FW−DW) × 100, where LWt is the weight of the thalli subjected to the desiccation treatment for t hours, dry weight (DW) is the weight of the thalli oven dried for 48 h at 80 °C, and FW is the weight of the thalli before desiccation.

### Determination of Reactive Oxygen Species (ROS)

ROS were determined according to the procedure described by Contreras *et al*.^40^. The thalli of *U*. *lactuca* (1 g FW) after different treatments of desiccation and submergence were incubated in 100 mL of 5 µM 2,4-dichlorofluoresceine diacetate (Calbiochem, San Diego, CA, USA) dissolved in filtered seawater for 1 h at 15 °C. After incubation, the thalli were rinsed in seawater, blotted dry, weighed, and frozen in liquid nitrogen. The tissues were then ground in liquid nitrogen, suspended in 5 mL of 40 mM Tris–HCl buffer, pH 7.0, and centrifuged at 8,000 × g for 25 min. Fluorescence was determined using LS-5 spectro-fluorometer (Perkin-Elmer, Norwalk, CT, USA) at an excitation wavelength of 488 nm and at an emission wavelength of 525 nm. Fluorescence values were obtained using a standard curve of 2,4-dichlorofluoresceine (DCF; Sigma, St. Louis, MO, USA).

### Histochemical localization of O_2_^•−^ and H_2_O_2_

The production of O_2_^•−^ and H_2_O_2_ in response to desiccation and re-submergence was analyzed according to Castro-Mercado *et al*.^41^. For the detection of O_2_^•−^ radicals, small section of algal thalli (20 in number) each for control, desiccated and re-submerged algae were immersed in 5 mL detection solution containing 0.05% nitroblue tetrazolium (NBT) in 50 mM potassium phosphate buffer (pH 6.4) and 10 mM sodium azide (NaN_3_). The sections were infiltrated under vacuum for 3 min in the same solution and illuminated for 2 h until the appearance of dark spots, characteristic of blue formazan precipitates. Stained sections were cleared by boiling in acetic:glycerol:ethanol (1:1:3, v/v/v) solution before photographs were taken.

H_2_O_2_ production was visually detected by an endogenous peroxidase-dependent staining procedure using 3,3-diaminobenzidine (DAB). Algal thalli (20 in number each) were immersed in DAB solution 1 mg/mL (pH 5.0), vacuum-infiltrated for 5 min and then incubated at room temperature for 8 h in the presence of light till brown spots appeared. Sectioned were bleached by immersing in boiling ethanol to visualize the brown spots and photographs were taken.

### Determination of lipid peroxidation

The level of lipid peroxidation in the thallus was determined as described by Heath and Packer^42^. The treated algal tissue (0.2 g) was extracted with 2 mL of 0.5% thiobarbituric acid (TBA) prepared in 20% trichloro acetic acid (TCA). The extract was heated at 95 °C for 30 min and then quickly cooled on ice. After centrifugation at 8,000 × g for 10 min, the absorbance of the supernatant was measured at 532 nm. Correction of non-specific turbidity was made by subtracting the absorbance value taken at 600 nm. The level of lipid peroxidation was expressed as nmol of malondialdehyde (MDA) formed using an extinction coefficient of 155 mM cm^−1^.

### Metabolomics using NMR spectroscopy

The untargeted metabolite profiling was performed using NMR spectroscopy following the method developed by Gupta *et al*.^9^. The aqueous extract of algal thalli after treatments of desiccation and submergence were prepared. The algal thalli were blotted on paper towel to remove excess saline water and then thalli of 200 mg fresh wt. were macerated using mortar and pestle. While maceration about 200 µL of 50 mM phosphate buffer adjusted to pH 6.0 was added. The aqueous extracts were vortexed for one minute and then sonicated for 30 min with heating at 55 °C. The extracts were then centrifuged at 10,000 rpm for 2 min and clear supernatant was collected. An aliquot of this solution was transferred directly to 5 mm NMR tube to which a few drops of D_2_O containing a reference standard (TSP) was added.

### NMR measurements and metabolite identification

NMR spectra were acquired on a Bruker Avance II 500 MHz spectrometer, equipped with a 5 mm BBI probe. Samples were spun at 20 Hz at temperature 20 °C. Each spectra was consisted of 80 scans of 2 s acquisition time with a spectral width of 7000 Hz. Spectra were Fourier transformed using an exponential line broadening value of 0.3 Hz. Spectra were then manually phased, baseline corrected and calibrated to the internal standard (TSP = 0.0 ppm). The solvent signal suppression was applied at 4.8 ppm during the recycle delay of 1 s with a low strength (bandwidth of 49 Hz) RF pulse. Ambiguities among the 1D pattern recognition were resolved by mapping the correlation among cross peaks of TOCSY (pre saturation version) and HSQC experiments. HSQC spectra were recorded by echo-antiecho method using gradients where 64 scans for 256 t_1_ points were acquired with recycle delay of 1.5 s. The total acquisition time was around 7 hrs. The broad residual water signal in HSQC spectra was removed by correcting the baseline with Gaussian function of width 0.3 ppm. TOCSY spectra were acquired with 24 scans for 128 t_1_ points. Metabolite identification was performed by comparing^1^H resonances and 2D correlation data against the reported literature and available online databases (http://prime.psc.riken.jp/, http://www.bmrb.wisc.edu/metabolomics/, http://www.hmdb.ca/), and Chenomx software (evaluation version).

### Data processing

The spectral data hereby generated was examined for relative intensity variations among the identified metabolites by scaling the peak intensity. NMR peaks were bucketed in the range of 0.0–10.0 ppm excluding the water region from 4.5–5.0 ppm using MestReNova (Mestrelab Research, Spain). The spectral peaks were then sum normalized for scaling.

### Statistical Analysis

All the experiments except NMR were performed with three biological replicates. The NMR experiments were carried out with two biological replicates. The data hereby presented is mean ± standard deviation (SD). The auto-scaled and log transformed metabolite profiles were analysed using the R and Bioconductor project^43^. Significantly present metabolites were selected using the significance level P < 0.05 as the cut-off after application of FDR correction for multiple testing. The heatmaps were plotted using pheatmap package of R.

## Electronic supplementary material

Supplementary Materials
