# Supplementary material for: Metabolic regulatory oscillations in intertidal green seaweed Ulva lactuca against tidal cycles
Source: Sci Rep. 2017 Nov 27;7:16430. doi: 10.1038/s41598-017-15994-2 (PMC5703862; doi:10.1038/s41598-017-15994-2)
Supplement: Supplementary file 1 — Supplementary Materials [file 41598_2017_15994_MOESM1_ESM.pdf]

Metabolic regulatory oscillations in intertidal green seaweed *Ulva lactuca* against tidal cycles

Vishal Gupta[a*], Hemant R Kushwaha[b#]

[a]Biological Oceanography Division, CSIR-National Institute of Oceanography, Goa-403004, India

[b]Synthetic Biology and Biofuel, International Centre for Genetic Engineering and Biotechnology, New Delhi, India 110067

Email: *vishalg@nio.org, #hemant@icgeb.res.in

Supplementary Table 1: Fold change variations in concentrations of metabolites at desiccation periods of 24 h, 36 h and 72 h with respect to submergence as control.

**Metabolite**	**Desiccation Duration**		
	**24 h**	**36 h**	**72 h**
Acetate	2.77623685	-1.92415292	-2.03299585
Alanine	1.10292606	-1.04490851	-1.40227102
Allantoin	-0.67295649	5.61427722	7.21216961
Ascorbate	-0.54264321	-2.4848137	-0.7419571
Betaine	1.96223493	-0.05072856	-0.26448409
Choline	8.10153183	0.28334482	0.36696821
Coniferaldehyde	2.38094952	0.48346647	0.08876555
Cysteine	1.26965914	-0.61316716	-0.48420605
Formic acid	-0.78539195	-1.6948562	-2.04284871
GABA	1.22691953	-1.42393764	-1.03689322
Glucose	0.44453446	-1.35746768	0.44557696
Glutamic acid	0.91906027	-2.96687079	-2.6804693
Glycine	1.59824843	-0.16264107	-0.52126146
Hypotaurine	1.61874627	0.022435	-0.10849421
Lactate	1.90734081	-0.27106787	-0.31258593
alpha-ketoglutaric acid	2.04218087	-2.08052886	-1.4450192
Sucrose	-1.31692635	4.0563983	5.09446235
Triethanolamine	5.76523808	-1.15453927	-1.04098518

Supplementary Table 2: Fold change variations in concentrations of metabolites at re-submergence periods of 0.5 h, 1 h and 6 h with respect to 24 h desiccation as control

**Metabolite**	**Desiccation**	**Re-submergence**		
	**24 h**	**0.5 h**	**1 h**	**6 h**
Acetate	2.776236845	-4.85684206	-4.67064986	-4.78937896
Alanine	1.102926057	-1.27754168	-1.033195	-0.7795282
Allantoin	-0.672956492	7.55237593	7.06837778	5.92822317
alpha-ketoglutaric acid	2.042180874	-3.84117801	-4.12490804	-4.86168128
Ascorbate	-0.542643213	-1.11808897	-1.30732995	-2.81191345
Betaine	1.96223493	-1.99790255	-1.97776633	-1.84934261
Choline	8.101531835	-7.70521291	-8.04969596	-7.58676052
Coniferaldehyde	2.380949521	-1.93462833	-1.99992912	-1.64443296
Cysteine	1.269659136	-2.01093185	-2.13163176	-2.35592985
Formic acid	-0.785391946	5.00984671	2.01772542	-1.77514436
GABA	1.226919528	-2.51746924	-2.70087559	-2.0441972
Glucose	0.444534462	-1.52359275	-1.38770872	-2.17221818
Glutamic acid	0.919060271	-3.88737772	-3.91707806	-3.36824344
Glycine	1.598248426	-2.0138221	-1.62392695	-1.67175054
Hypotaurine	1.618746272	-1.96045641	-1.85765281	-1.80856374
Lactate	1.90734081	-2.20613481	-1.90661595	-2.12035612
Sucrose	-1.316926353	5.75675227	4.95101204	6.13429534
Triethanolamine	5.765238078	-6.4777305	-6.61336775	-5.38601307

Supplementary Table 3: Fold change variations in concentrations of metabolites at re-submergence periods of 0.5 h, 1 h and 6 h with respect to 36 h desiccation as control

**Metabolite**	**Desiccation**	**Re-submergence**		
	**36 h**	**0.5 h**	**1 h**	**6 h**
Acetate	-1.92415292	0.43143892	0.16601938	0.77816345
Alanine	-1.04490851	-0.07954517	1.59702026	1.06769557
Allantoin	5.61427722	0.92622763	-0.37205982	-1.7641732
alpha-ketoglutaric acid	-2.08052886	0.37746097	-0.42810916	-0.4414098
Ascorbate	-2.4848137	0.60724892	4.07137312	0.67854879
Betaine	-0.05072856	-0.24429976	-0.04102297	-0.07399492
Choline	0.28334482	0.25250755	0.71336344	2.88174365
Coniferaldehyde	0.48346647	-0.38874709	0.14050145	0.35469463
Cysteine	-0.61316716	-0.11505013	-0.5124492	-0.46036631
Formic acid	-1.6948562	-0.64815531	-0.56785083	-0.91321785
GABA	-1.42393764	0.97382375	0.83766262	5.30748067
Glucose	-1.35746768	0.72502936	3.46749782	0.91662845
Glutamic acid	-2.96687079	0.7303236	0.71413778	1.50391615
Glycine	-0.16264107	-0.22016648	-0.23088744	-0.30492873
Hypotaurine	0.022435	-0.00056287	-0.43660174	-0.52665873
Lactate	-0.27106787	-0.18268645	0.36935804	0.13480648
Sucrose	4.0563983	1.66720866	-0.22685285	-1.71068318
Triethanolamine	-1.15453927	0.24121234	0.99275112	5.16222722

Supplementary Table 4: Fold change variations in concentrations of metabolites at re-submergence periods of 0.5 h, 1 h and 6 h with respect to 72 h desiccation as control.

**Metabolite**	**Desiccation**	**Re-submergence**		
	**72 h**	**0.5 h**	**1 h**	**6 h**
Acetate	-2.03299585	0.61366067	0.13220343	0.80162954
Alanine	-1.40227102	0.29142548	0.56755905	1.81999833
Allantoin	7.21216961	-0.46018893	0.55042565	-1.38837723
alpha-ketoglutaric acid	-1.4450192	-0.2561437	-1.14363618	-1.27891733
Ascorbate	-0.7419571	-1.00844603	-1.51969766	-2.55871961
Betaine	-0.26448409	0.00188412	0.21441132	0.14167844
Choline	0.36696821	0.48011239	0.51922606	0.36787411
Coniferaldehyde	0.08876555	0.08935675	0.30972498	0.54490067
Cysteine	-0.48420605	-0.27903376	-0.57181826	-0.59520412
Formic acid	-2.04284871	-2.28836734	0.99516017	-0.31759934
GABA	-1.03689322	1.11598137	0.31661762	1.58462988
Glucose	0.44557696	-0.76995158	0.86696684	-2.23446978
Glutamic acid	-2.6804693	0.51914509	0.15007461	0.86596309
Glycine	-0.52126146	0.13785182	0.29770949	0.23261618
Hypotaurine	-0.10849421	0.1435207	0.27979129	-0.16599982
Lactate	-0.31258593	-0.11116828	0.30814193	-0.1540441
Sucrose	5.09446235	3.07805505	0.07758819	3.13580818
Triethanolamine	-1.04098518	0.31334557	0.70859149	0.6279014

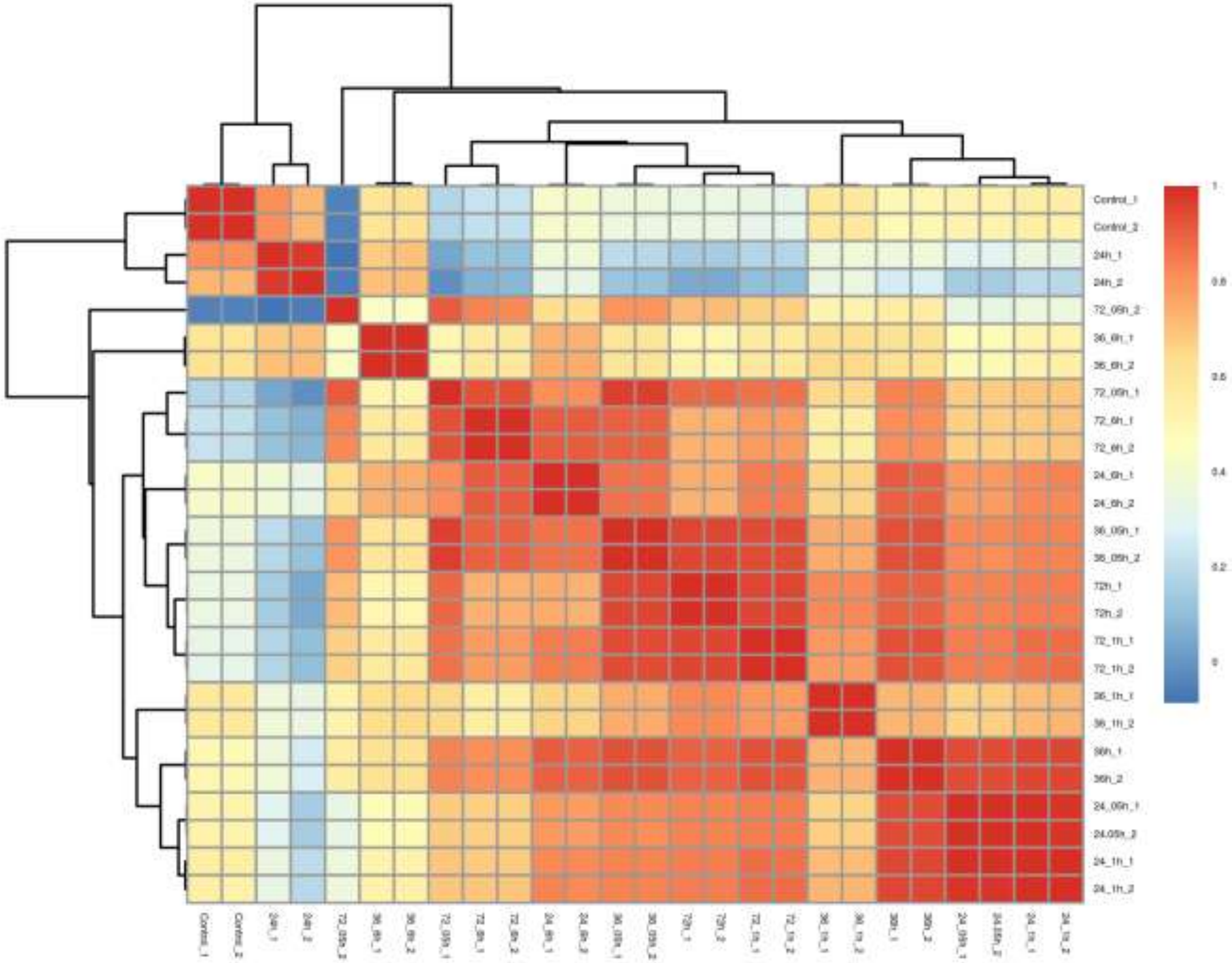

Supplementary Figure 1: Heatmap representation of the correlation among the experimental conditions of different desiccation and re-submergence time periods.
